# The posterior communicating arteries in the patients with sudden deafness: evaluation with magnetic resonance imaging (MRA)

**DOI:** 10.1186/1472-6815-6-5

**Published:** 2006-03-21

**Authors:** Yoshito Tsushima, Ayako Taketomi-Takahashi, Keigo Endo

**Affiliations:** 1 Department of Diagnostic Radiology and Nuclear Medicine, Gunma University Hospital, 3-39-15 Showa-machi, Maebashi, Gunma 371-8511 Japan; 2 Department of Radiology, Motojima General Hospital, 3–8 Nishi-Honcho, Ohta, Gunma 373-0033 Japan

## Abstract

**Background:**

A strong association was suggested between a non-functioning posterior   communicating artery (Pcom) of the circle of Willis and sudden deafness   (SD). The purpose of this study was to determine the rate of depiction of   Pcom on magnetic resonance angiography (MRA) in patients with SD.

**Methods:**

Sixteen patients with SD (47.7 +/- 13.3 years; range, 24 – 76 years; nine males) were evaluated with intracranial MRA as well as magnetic resonance imaging (MRI) of the head. The depiction of Pcom on MRA was correlated with the laterality of SD. One hundred twenty-eight controls (49.1 +/- 8.4 years; range, 22 – 66 years; 87 male) were selected from neurologically normal subjects who underwent MR examinations as a part of an annual medical check-up in our hospital.

**Results:**

Four (25%) of 16 SD patients had bilateral Pcom on MRA, four patients had unilateral Pcom and eight patients had bilaterally absent Pcom These results were not significantly different from the controls (p = 0.96). In 6 (37.5 %) of 16 SD patients, the ipsilateral Pcom was present on MRA, and 104 (40.6%) of 256 Pcom were present in 128 normal controls (p = 0.81).

**Conclusion:**

Since there was no link between the occurrence of SD and the absence of the ipsilateral Pcom, our results cannot support the hypothesis that the absence of Pcom may be a risk factor for the occurrence of SD.

## Background

Sudden deafness (SD) accounts for approximately 1% of all cases of sensorineural hearing loss, and most cases of spontaneous SD have no identifiable cause [1]. Numerous clinical and laboratory investigations have attempted to identify the case of this disorder, and although the suggested causes include circulatory disorders, viral infections, and labyrinthine trauma, there is no definitive proof.

Recently, De Felice et al. [2] took ultrasonographic doppler flow measurements of the extracranial carotid and vertebrobasilar systems and independent audiological measurements in SD patients, and suggested a strong association between a non-functioning posterior communicating artery (Pcom) of the circle of Willis and SD. Although the mechanisms for the observed association remain unknown, their observations may be consistent with a circulatory pathogenesis of idiopathic SD.

We performed magnetic resonance imaging (MRI) with and without contrast material and magnetic resonance angiography (MRA) of the circle of Willis in 16 patients with SD and 48 controls subjects matched for age and sex in order to determine the possible correlation between the absence of Pcom and SD.

## Methods

In our hospital all patients with SD undergo enhanced MRI of the head and MRA of the circle of Willis in order to depict possible etiology of SD, in particular cerebellopontine angle tumors. During a period of one year, sixteen patients with unilateral SD (mean +/- standard deviation, 47.7 +/- 13.3 years; range, 24 – 76 years; nine males) were evaluated with intracranial MRI and MRA, and all of these patients were included in this study. By definition, SD is rapid (over a period of up to three days) sensorineural hearing loss. Hearing loss was defined as a hearing level loss of 30 dB or more in at least three contiguous audiometric frequencies. One patient was diabetic, but none of the 16 patients were hypertensive. There was no evidence of cranial nerve abnormalities except for the eighth nerve, and general physical examinations were otherwise normal. No patients had any history of trauma, intense noise exposure, or recent viral illness, which may result in sensorineural hearing loss. Three patients had vertigo and one had tinnitus. These medical evaluations were all performed in the otorhinolaryngology department of our hospital.

All subjects were studied on the same 1.0-T MR scanner (Magnex^®^, Shimadzu, Kyoto, Japan), including axial T1-weighted spin echo (SE; TR = 450 ms, TE = 15 ms), and proton-density-weighted and T2-weighted fast SE (TR = 4000 ms, TE = 20 and 100 ms, echo train length = 8). The matrix was 256*192 and the section thickness was 5 mm with a gap of 2.5 mm. MRA was also performed using time-of-flight technique (TR = 40 ms, TE = 9 ms, flip angle = 20°), and 16 projections of the MRA of the circle of Willis were created by a maximum-intensity projection (MIP) algorithm around the head-to-foot axis and right-to-left axis. Coronal and axial T1-weighted images with contrast enhancement (TE = 450 ms, TE = 15 ms) were also obtained in all subjects by intravenous administration of Gd-DTPA (Magnevist^®^, Nihon Schering, Osaka, Japan). The matrix was 256*192 and a section thickness was 3 mm with a gap of 1 mm. All MR images were interpreted by a board-certified diagnostic radiologist, and all MR abnormalities were recorded. MRA findings were also recorded, and the particular attention was paid for the presence of the Pcom.

The control subjects consisted of 128 patients (49.1 +/- 8.4 years; range, 22 – 66 years; 87 male) in whom no important abnormal findings were observed on MRI and MRA. They were selected from subjects who underwent MR examinations, which were performed during the same period of the SD subjects, as a part of an annual medical check-up in our hospital. They were neurologically healthy, no cochlear or vestibular symptoms were present, and normal standard pure tone audiometry was within normal limits. The protocol of MRI and MRA examinations was the same as that for SD patients, although no enhanced T1-weighted images were obtained.

Student t test and Fisher's exact test were employed for statistical analyses, and a p value of 0.05 or less was considered significant. In our hospital, no approval of the ethics committee was necessary for this kind of a retrospective study. The Declaration of Helsinki principles was followed.

## Results

**Figure 1:**
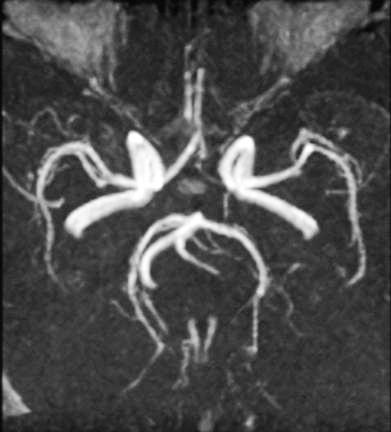
**MRA of a 47-year-old male patient with right SD. **The right Pcom was absent, but the left one (arrow) is clearly demonstrated.

Four (25%) of 16 SD patients had bilateral Pcom on MRA, four patients had a unilateral Pcom and eight patients had a bilaterally absent Pcom (Figure 1). These results were not significantly different from that of the controls (p = 0.96; Table 1). Correlation between the presence of the Pcom and the side of SD is shown in Table 2. In 6 (37.5 %) of 16 SD patients, the ipsilateral Pcom was present on MRA, and 104 (40.6%) of 256 Pcom were present in 128 normal controls (p = 0.81).

**Table 1:** Posterior communicating artery (Pcom) in SD patients and controls. There was no association between the type of subject and the type of Pcom (p = 0.96, Fisher's exact test).

	**Posterior communicating artery (Pcom)**			
	**Bilaterally present**	**Unilaterally present**	**Bilaterally absent**	**Total**

**SD patients**	4 (25%)	4 (25%)	8 (50%)	16
**Controls**	35 (27.3%)	34 (26.6%)	59 (46.1%)	128

**Total**	39	38	67	144

**Table 2:** Ipsilateral posterior communicating artery (Pcom) in SD patients and controls. There was no association between the type of subject and the presence of ipsilateral Pcom (p = 0.81, Fisher's exact test).

	**Ipsilateral Posterior communicating artery (Pcom)**		
	**Present**	**Absent**	**Total**
**SD patients**	6 (37.5%)	10 (62.5%)	16
**Controls**	104 (40.6%)	152 (59.4%)	256 *

* Two hundreds fifty-six Pcom in 128 control subjects.

In the SD patients and controls, no vascular abnormalities, such as aneurysm and arterial stenosis, were observed on MRA. In one patient with SD, a small arachinoid cyst in the middle cranial fossa was demonstrated on MRI. Otherwise, on MRI with and without contrast enhancement, no abnormal findings, such as tumor, infarction and degenerative diseases, were observed in either the SD patients or controls. There was no differences between the SD patients and control subjects in age (p = 0.69) and sex (p =   0.35).

## Discussion

Pcom is a potential collateral pathway through which adequate distribution of cerebral blood flow can be maintained in case of impaired of decreased flow through one of more of its proximal feeding vessels, and a small or absent ipsilateral Pcom is a risk factor for ischemic cerebral infarction in patients with internal carotid artery occlusion [3,4]. It has been also reported that the symptom complex of vertebrobasilar insufficiency can be correlated with the absence of Pcom [5]. From the viewpoint of a circulatory pathogenesis of SD, the absence of Pcom might also contribute to the occurrence of SD [2]. However, our current data did not demonstrate any correlation between an absent Pcom and the development of SD, and thus failed to support the hypothesis that the absence of Pcom may be a risk factor for the occurrence of SD.

According to normal reference values for the presence of the anatomic variants of the circle of Willis on MRA using 1.5-T unit [4], the prevalence of an entirely complete configuration of the circle of Willis is only 54%. Hoksbergen et al. [6] compared MRA findings to transcranial color duplex sonography, and concluded that if Pcom can be visualized by MRA, it can be assumed with a high level of confidence that collateral flow is possible. In our study, the prevalence of the presence of ipsilateral Pcom in SD patients was 37.5%, and this prevalence was not significantly different compared to the normal controls (40.6%).

Since our MR unit was 1.0-T, the spatial resolution may be slightly lower than a 1.5-T unit. Therefore, some very small Pcoms might not be depicted on MRA, possibly resulting in a higher false negative rate. In a previous study using 1.5-T MR system [6], the posterior collateral pathway was judged nonfunctional on MRA in 31% of all cases, and this prevalence was slightly lower compared to our results (59.4%). However, even if Pcoms with a very small diameter were depicted on MRA, the overall result in our study would not change.

Our examined population was Japanese and De Felice et al. [2] studied a Caucasian population. This difference may be the reason for the different results, but to our knowledge, there have been no reports showing racial differences of Willis anatomy.

In spite of our results, circulatory disorders may still be a possible cause of SD. If only a small percentage of SD is due to circulatory disorders, it would not be surprising that no statistically significant difference in the absence of Pcom can be observed. More detailed analyses with a large number of SD patients are encouraged to elucidate the possible circulatory pathogenesis of SD.

## Conclusion

Since there was no link between the occurrence of SD and the invisible Pcom on MRA, our results cannot support the hypothesis that the absence of Pcom may be a risk factor for the occurrence of SD.

## List of abbreviations

MIP: maximum-intensity projection

MR: magnetic resonance

MRA: magnetic resonance (MR) angiography

MRI: magnetic resonance (MR) imaging

Pcom: posterior communicating artery

SD: sudden deafness

SE: spin echo

TE: echo time

TR: repetition time

## Competing interests

The author(s) declare that they have no competing interests.

## Authors' contributions

YT participated in the design of the study and performed the statistical analysis. KE conceived of the study, and participated in its design and coordination and helped to draft the manuscript. Both authors read and approved the final manuscript.

## Pre-publication history

The pre-publication history for this paper can be accessed here:


